# Clearing the air: Addressing air clearance times for infectious aerosols in healthcare facilities

**DOI:** 10.1017/ice.2020.432

**Published:** 2020-08-24

**Authors:** Andrew W. Hurlburt, Rita J. DeKleer, Elizabeth A. Bryce

**Affiliations:** Divisions of Medical Microbiology and Infection Prevention, Vancouver Coastal Health, Vancouver, British Columbia

*To the Editor—*Recently, the issue of air clearance of COVID-19 following aerosol-generating medical procedures (AGMPs) has become a source of debate and angst among clinicians and infection preventionists. Infection prevention, surgical, and anesthesiology societies have estimated COVID-19 air clearance times based on a table published in a 2003 Centers for Disease Control *Morbidity and Mortality Weekly Report* that lists the number of air exchanges per hour required to clear varying fractions of aerosolized tuberculosis particles. This has dictated the length of time operating rooms are furloughed before subsequent cases can proceed without the use of a particulate respirator, when transport to a postanesthesia recovery area can occur, and when patient rooms can be put back in circulation.^1,2^ Based on this approach, a room could be closed to new procedures or admissions for between 30 minutes and >2 hours. As hospitals return to the “new normal” and begin addressing their backlog of cases, these recommendations will result in increased turnaround times for procedures and patient admissions at a critical juncture for clinical care.

Few realize that the CDC table was originally derived from a mathematical formula presented in a 1973 NIOSH publication by Mutchler^3^ on controlling the industrial environment. Heating, ventilation, and air conditioning (HVAC) systems have since become more sophisticated, and factors such as relative humidity, percent recirculated air, placement of exhaust, traffic flow, dilutional airflow, percent recirculated air, and room clutter have been recognized as additional important determinants in the removal of infective particles. Both the American Society of Heating, Refrigerating, and Air-Conditioning Engineers (ASHRAE) and The American Society for Healthcare Engineering (ASHE) have recently highlighted the importance of these variables.^4,5^

Relative humidity in particular has been identified as a key determinant in reducing infectivity of aerosolized infectious particles. Noti et al^6^ reported that the retained infectivity of aerosolized influenza A was only 15%–22% at a relative humidity >40% compared to an infectivity of >70% at a relative humidity <20%, with most viral inactivation occurring within minutes of aerosolization. This finding was consistent across all particle sizes, and was most prominent in particles <1 µm in diameter, the particle size that predominates in bioaerosols.^6^ Yang and Marr^7^ also showed that a relative humidity of 50%–90% destabilizes multiple other viruses, including SARS coronavirus. Furthermore, recent epidemiological data suggest that transmission of COVID is less efficient in warm humid environments; researchers have theorized that increased relative humidity improves host factors such as mucociliary clearance of pathogens, intercellular interferon signaling, and tissue repair.^8^

**Fig. 1.**
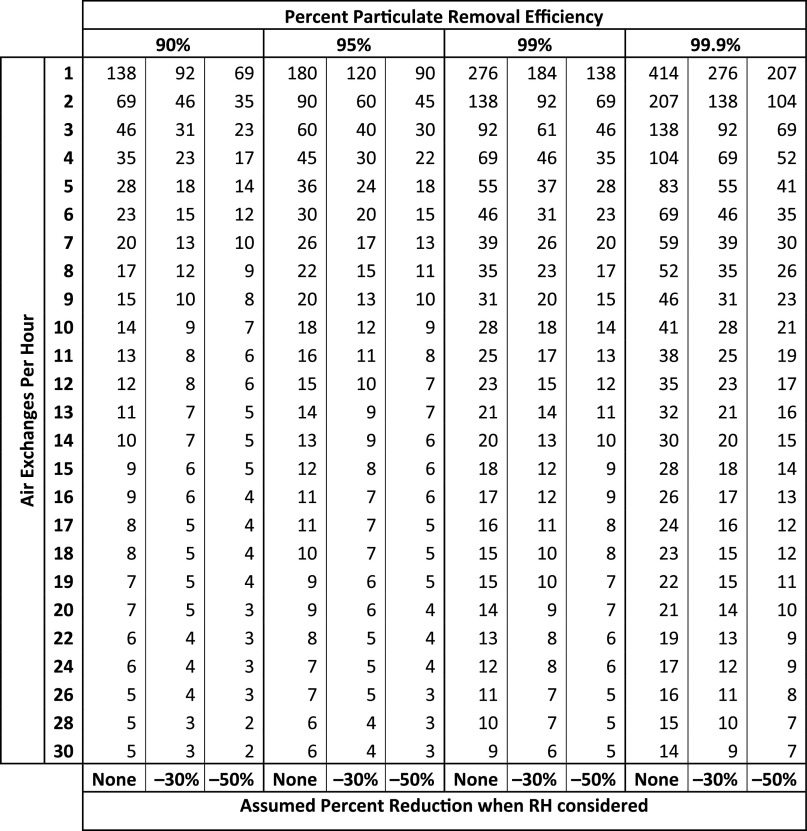
Comparison of times required to clear particulates: The effect of air exchanges alone versus air exchanges and a relative humidity of between 40% to 60%. The following formula was used to estimate air clearance times (in minutes) for set Percent Particulate Removal Efficiencies (PRE) with changing Air Exchange Rates per hour (ACH) and both conservative (−30%) and moderate (−50%) reduction factors (RF) to account for a relative humidity (RH) of between 40 and 60%:
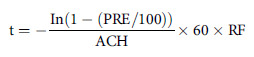

Taking these factors into consideration, our team of surgeons, anesthesiologists, and infection preventionists developed a pragmatic approach for estimating the time required to remove and/or inactivate aerosolized infectious particles. Figure 1 compares the air clearance times sufficient to remove 90%–99.9% of bioaerosols at varying air exchange rates from the aforementioned CDC table to modified times that reflect additional 30% (conservative estimate) and 50% (moderate estimate) reductions in infective particles to account for the effect of relative humidity between 40% and 60% This relative humidity is achievable in most interventional radiology suites, operating rooms, and many patient rooms. It is also consistent with the current ANSI/ASHRAE/ASHE Standard 17022008 recommendation for relative humidity in critical-care areas.

The time required for a removal efficiency of 95% (conceptually in alignment with the particulate efficiency of a fit-test respirator) was established as a reasonable end point that balanced both patient and healthcare worker safety. The concept that a 95% removal efficiency was safe resonated with and was accepted by physicians based on 2 factors. First, despite technical differences between 95% removal efficiency and the 95% filtration efficiency of a N95-rated respirator, which applies to particles 0.3 µm or larger in diameter, it was felt that these would be equivalent by the time air clearance time had elapsed, due the fact that larger particles would settle quickly with gravity. Furthermore, any residual particles, regardless of size, would have a significantly decreased infectivity due to relative humidity.^6^ To further improve air clearance, there was commitment to minimize traffic and equipment in the suites to optimize air circulation and minimize cross transmission on surfaces. Although the incremental benefit of any one of these additional measures was unknown, it was felt that all would act to increase the margin of safety.

Using the traditional approach of air exchanges alone, a room with 15 air exchanges per hour would require 28 minutes to clear 99.9% of airborne particles. By targeting 95% air clearance and using a conservative reduction in viral activity of 30% at a relative humidity of between 40% to 60%, a room would be deemed safe for reoccupancy at 8 minutes. Moreover, procedure rooms in older hospitals with 10 air exchanges per hour would require furlough periods of only 12 minutes between patients versus 41 minutes according to previous recommendations. These modified times also approximate the duration required to prepare a postintervention patient for the recovery room, after which personnel would no longer require respiratory precautions in the absence of ongoing AGMPs.

The interaction between viruses and relative humidity is complex, and large knowledge gaps exist. Further research into the role of the built environment in management of bioaerosols in healthcare is required and will aid in the revision of national and international infection prevention guidelines. Until knowledge in this area is further advanced, we hope others will find this a rational and practical approach that balances healthcare worker and patient safety while preserving patient flow in areas where AGMPs are commonly performed.
